# The development status of PET radiotracers for evaluating neuroinflammation

**DOI:** 10.1007/s13139-023-00831-4

**Published:** 2024-01-08

**Authors:** Namhun Lee, Jae Yong Choi, Young Hoon Ryu

**Affiliations:** 1 Division of Applied RI, Korea Institute of Radiological & Medical Sciences, 75 Nowon-ro, Nowon-gu, Seoul, 01812 Korea; 2 Radiological and Medico-Oncological Sciences, University of Science and Technology (UST), Seoul, Korea; 3 Department of Nuclear Medicine, Gangnam Severance Hospital, Yonsei University College of Medicine, Seoul, Korea

**Keywords:** Neuroinflammation, Microglia, Astrocyte, Positron emission tomography, Radiotracer

## Abstract

Neuroinflammation is associated with the pathophysiologies of neurodegenerative and psychiatric disorders. Evaluating neuroinflammation using positron emission tomography (PET) plays an important role in the early diagnosis and determination of proper treatment of brain diseases. To quantify neuroinflammatory responses in vivo, many PET tracers have been developed using translocator proteins, imidazole-2 binding site, cyclooxygenase, monoamine oxidase-B, adenosine, cannabinoid, purinergic P2X7, and CSF-1 receptors as biomarkers. In this review, we introduce the latest developments in PET tracers that can image neuroinflammation, focusing on clinical trials, and further consider their current implications.

## Introduction

Neurodegeneration is characterized by gradual and progressive loss of the neuronal population, whereas psychiatric disorders are characterized by the disruption of neurocircuits in the central nervous system (CNS) [1]. These brain diseases are closely related to age, and their prevalence tends to increase as society ages; such affect not only patients themselves but also other’s lives. Therefore, diagnosing brain diseases early is crucial. Although decades of efforts developing therapeutic drugs to overcome brain diseases, most clinical trials have failed, and currently prescribed drugs are used for temporary relief and to slow disease progression [2, 3]. To understand the mechanisms underlying the pathophysiology of brain diseases, considering not only the damage to certain brain tissues but also the surrounding neuronal environment is necessary.

Neuroinflammation is an innate immunological response in the CNS activated in response to various pathological insults, including infection, misfolded protein aggregation, and trauma [4]. Microglia and astrocytes, the main immune components, are key regulators of neuroinflammation and play crucial homeostatic roles for neuronal functioning [5]. Under physiological conditions, these cells maintain homeostasis by removing cellular debris during phagocytosis. However, in pathological conditions caused by chronic insults beyond their threshold, such glial cells become overactive and release various neurotoxins that induce neuronal death. The neurotoxins in turn activate glial cells again, destroying the continuous neural circuit in a vicious cycle [6]. Neuroinflammation plays an important role in various neurodegenerative diseases and psychiatric disorders, including Alzheimer’s disease (AD), Parkinson’s disease (PD), amyotrophic lateral sclerosis (ALS), major depressive disorder (MDD), and posttraumatic stress disorder (PTSD) [7].

Positron emission tomography (PET) is a molecular imaging technique that can evaluate in vivo biochemical changes in living organisms without pharmacological effects, allowing the functional evaluation of neuroinflammatory responses in a noninvasive manner [8]. Elucidating the dynamic roles of glial cells in disease progression with PET is useful in the determination of disease status or evaluation of therapeutic intervention, as inflammatory reactions occur before the clinical symptoms of brain diseases are expressed. Multiple lines of evidence suggest that inflammation and reactive gliosis occur years before the onset of AD [9–11].

**Fig. 1:**
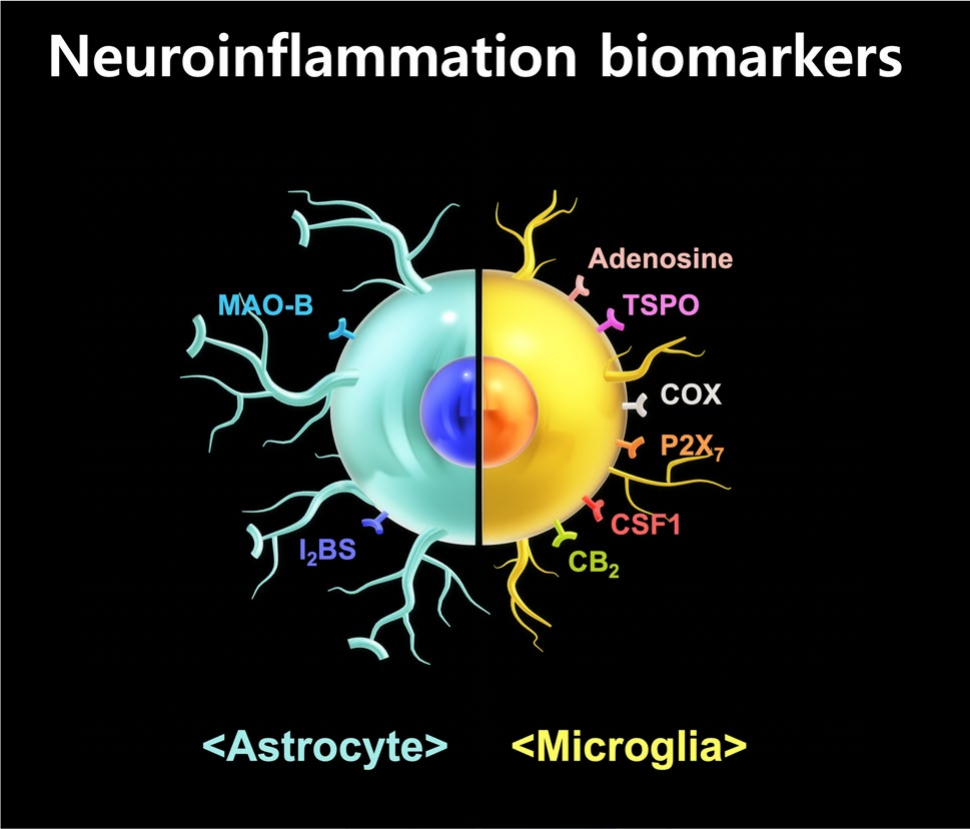
Types of microglia and astrocyte-based biomarkers for evaluating neuroinflammation

As illustrated in Fig. 1, representative biomarkers for neuroinflammation can be largely divided into proteins (translocator proteins), enzymes (cyclooxygenase and monoamine oxidase-B), and various receptors (imidazole-2 binding site, and adenosine, cannabinoid, purinergic P2X7, and colony stimulating factor-1 receptors). Although research began approximately 40 years ago, attempts to assess neuroinflammation have yet to obtain robust results in clinical trials. In the present review, we introduce the state-of-the-art status of the development of PET tracers that can evaluate neuroinflammation and consider their implications at this point in time.

## Imaging Microglia

### Translocator Proteins (TSPO)

The first PET tracer developed to assess microglial activity through translocator proteins (TSPO) was [^11^C]PK11195, which showed increased binding in patients with AD compared with healthy controls [12]. In a head-to-head comparison study with [^11^C]PK11195 and [^11^C]PIB (a specific radiotracer for amyloid-β plaques), the [^11^C]PK11195 binding was increased in the cortical and sub-cortical areas in which uptake was increased in [^11^C]PIB, and an inverse correlation with the mini-mental state exam (MMSE) score, but not with the [^11^C]PIB binding ratio [13]. These results indicated that a neuroinflammatory response may occur prior to amyloid pathology. However, [^11^C]PK11195 has limitations such as low permeability and high non-specific plasma binding [14].

To overcome the shortcomings of [^11^C]PK11195, second-generation TSPO PET radiotracers, including [^11^C]DAA1106, [^11^C]DPA713, [^18^F]PBR06, [^18^F]PBR28, and [^18^F]DPA714 have been developed [15]. [^3^H]DAA1106 showed a tenfold higher binding affinity and approximately 1.5 times higher specific binding than [^3^H]PK11195 in tissues of individuals with neurological diseases compared to controls [16]; however, it failed to differentiate a rat model of herpes encephalitis from a control group [17]. PET employing [^11^C]DPA713 presented a lower background signal and higher specific binding than [^11^C]PK11195 in a rat model of herpes [18]. Yokokura et. al. reported that [^11^C]DPA713 PET showed increased uptake in the brain of patients with AD compared to healthy subjects. However, [^11^C]PK11195 PET displayed no difference in individuals with AD, and [^11^C]DPA713 binding values were also significantly negatively correlated with MMSE [19]. These results imply that [^11^C]DPA713 is more sensitive to TSPOs than [^11^C]PK11195. In a multiple sclerosis (MS) study using [^18^F]PBR06, the uptake ratio in the right striatum was elevated in patients with MS with or without fatigue [20]. In patients with early phase PD, [^18^F]PBR06 showed an increased brain uptake ratio in the ipsilateral putaminal side of the motor onset, and these binding ratios were correlated with dopaminergic denervation but not with disease severity [21]. In a study on TSPO changes in AD, Kreisl et al. demonstrated that the binding of [^11^C]PBR28 increased in the cortical region, especially in the parietal and temporal lobes, compared to the control group, and that the binding was inversely proportional to cognitive function. This increase in binding was more significant in early onset than in late onset AD [22]. In a study on lateral sclerosis using [^11^C]PBR28, brain uptake was increased in amyotrophic and primary lateral sclerosis [23]. An important drawback of second-generation TSPO radiotracers is their large inter-subject variability due to a single nucleotide polymorphism in rs6971 or rs2997325, which generates high-, mixed-, and low-affinity binders between subjects [24–28]; here, a “low-affinity binder” indicates that the radiotracer’s binding to a specific target biomarker (i.e., TSPO) is low, which greatly degrades the quality of PET images. Polymorphisms in subjects can be identified through genetic analysis of leukocytes, and the results can be used to interpret PET images from second-generation PET tracers.

Third-generation PET radiotracers, including [^11^C]ER176, [^18^F]GE180, and [^18^F]BS224, have been developed to minimize the effects of genetic polymorphisms. A comparative study in healthy volunteers reported that [^11^C]ER176 was more stable in arterial blood and possessed a higher distribution volume and binding potential than [^11^C]PBR28 [29]. In clinical applications, [^18^F]GE180 showed strong tumor uptake in patients with untreated and pretreated glioblastoma (maximum tumor-to-background ratio, 6.61) [30]. [^18^F]GE180 presented high uptake, determining active MS lesions, in patients with relapsing–remitting MS regardless of individual binding status [31]. [^18^F]GE180 displayed increased brain uptake in cerebral cavernous malformations [32]. In an in vitro competitive inhibition assay, [^11^C]ER176 and [^18^F]GE180 were reported to be minimally affected by polymorphisms; however, these PET tracers displayed a considerable decrease in low-affinity binders [33, 34]. [^18^F]BS224 has recently been developed as a PET tracer that is insensitive to TSPO polymorphisms, and its efficacy has been proven in a lipopolysaccharide (LPS)-based inflammatory model; however, the results of its clinical application studies are still unknown [35].

Although TSPO-specific PET tracers are the most studied biomarkers of neuroinflammation, they are localized in the outer mitochondrial membrane and are mainly expressed in microglia and astrocytes [36]. Therefore, as determining whether the increased PET signal through TSPO is caused by microglia, astrocytes, or both is not possible, other biomarker-based PET tracers with high selectivity for microglia have been developed.

### Adenosine Receptor

Adenosine is an endogenous purine nucleoside derived from the hydrolysis of adenosine triphosphate. In a pathological state, neurons and glial cells release adenosine into the extracellular space, promoting inflammation that exacerbates neuronal injury [37]. Adenosine receptors are expressed on astrocytes and microglia [38]. To date, PET studies evaluating adenosine receptors have mainly been conducted using adenosine receptors 1 (A_1_) and 2 (A_2_). [^11^C]MPDX and [^18^F]CPFPX were developed as selective PET radiotracers for A_1_ receptors, whereas [^11^C]TMSX, [^11^C]SCH442416, [^18^F]MNI-444, and [^11^C]preladenant were developed for A_2_ receptors.

In early drug-naïve patients with PD, [^11^C]MPDX PET did not reveal altered A_1_ receptors compared with healthy controls [39]; however, an increased binding potential was identified in the frontal cortex, posterior cingulate cortex, and Rolandic area in patients with traumatic brain injury [40]. [^18^F]CPFPX PET showed increased uptake in ischemic hemispheres, followed by a progressive decline [41]; the radiotracer presented a relatively low fraction of specific binding (total equilibrium uptake: 33% and 66% in the cerebellum and cortex, respectively) in a human displacement study, resulting in difficulties in performing kinetic modeling using the cerebellum as a reference region [42].

In a clinical study on [^11^C]TMSX, Mishina et al. demonstrated increased putaminal binding in patients with PD with dyskinesia, but similar striatal binding between drug-naïve patients with PD and healthy controls [43]. [^11^C]TMSX PET showed increased brain uptake in a study of secondary progressive MS [44]. [^11^C]SCH442416 PET revealed that patients with PD with levodopa-induced dyskinesia possessed higher binding values than those without dyskinesia, who had comparable binding potential to healthy controls [45]. [^18^F]MNI-444 PET showed reasonable binding values (binding potentials: 2.6–4.9 in A_2_-rich regions) and good test-retest variability (<10%) in a preliminary human study [46]; however, a clinical application study using [^18^F]MNI-444 has not yet been conducted. Employing the [^11^C]preladenant, PET generated similar basal ganglia binding between the PD and control groups [47].

### Cannabinoid Receptor 2 (CB_2_)

The cannabinoid receptor 2 (CB_2_) is primarily expressed on the peripheral immune cells [48]. In the brain, CB_2_ expression is low and mainly found in microglia [49]. In an inflammatory state, CB_2_ expression is upregulated depending on the inflammatory context [50]. The radiochemistry of [^11^C]methoxy-Sch225336 was established, however, its brain uptake was low, possibly due to efflux pumps in the blood-brain barrier [51]. The [^11^C]NE40 demonstrated specific binding to CB_2_ in a rat model with local overexpression of the human CB_2_ receptor [52]. However, in the first-in-human study, [^11^C]NE40 PET presented a lower receptor availability in patients with AD, and did not differentiate patients with AD from healthy controls [53]. The reason for the failure in clinical patient application was thought to be the low affinity for CB_2_, and subsequent studies were conducted to improve the binding affinity.

Although [^11^C]A-836339 greatly improved the binding affinity for CB_2_ compared to [^11^C]NE40 (Ki 0.7 nM and 9.6 nM for [^11^C]A-836339 and [^11^C]NE40, respectively), no significant was identified difference between stroke or ischemic animal models and sham operated ones [54–56]. [^11^C]GW405833 showed a high affinity for CB_2_ (Ki 3.6 nM) and 78-fold selectivity for rats’ CB_1_ [57]. However, it presented a slow washout and high non-specific binding in a nonhuman primate model [58]. [^18^F]MA3 with 62 times greater affinity for CB_2_ and 13 times greater selectivity than [^11^C]NE40, has been developed [59]; in a subsequent study, [^18^F]MA3 showed specific binding in a rat model with local overexpression of the CB_2_ receptor; however, no difference was found between the control and blockade in a healthy nonhuman primate [60].

### Cyclooxygenase (COX)

Arachidonic acid (AA) is a polyunsaturated fatty acid found in the cell membrane phospholipids. If tissue injury occurs, the phospholipid membrane produces AA by phospholipases A_2_ and C. Then, cyclooxygenase (COX) catalyzes the conversion of AA to prostaglandins G_2_ and H_2_, which are related to neuroinflammation [61]. COX-1 and COX-2 are expressed in microglia and neurons in the CNS and are involved in neuroinflammation [62].

**Fig. 2:**
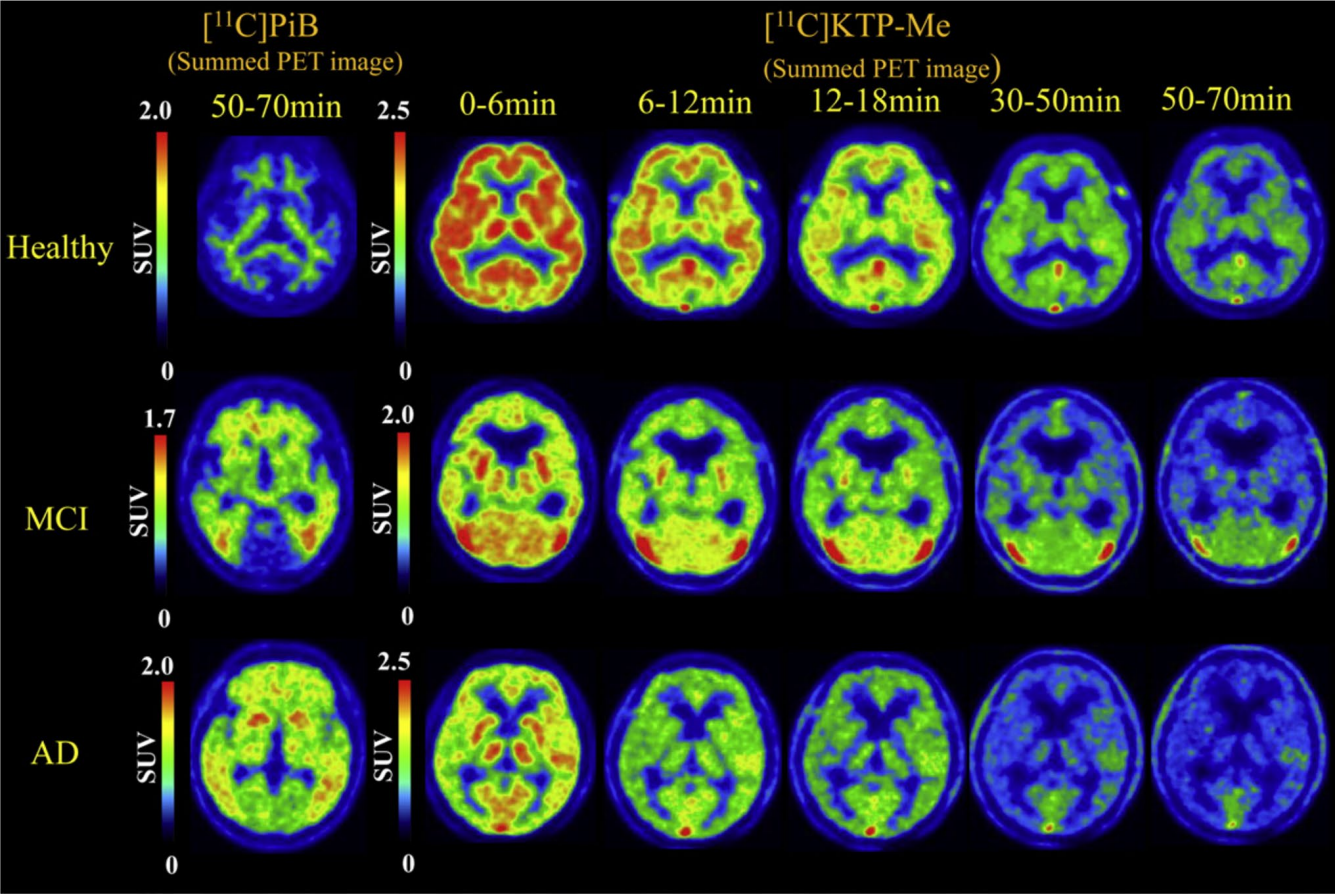
Comparison of [^11^C]PIB and [^11^C]KTP-Me in Alzheimer’s disease. Reprinted from “Exploratory human PET study of the effectiveness of ^11^C-ketoprofen methyl ester, a potential biomarker of neuroinflammatory processes in Alzheimer's disease”, volume 43, Ohnishi A, Senda M, Yamane T, Mikami T, Nishida H, Nishio T, et al. Nuclear Medicine and Biology, 438-444, Copyright (2016), with permission from Elsevier

[^11^C]KTP-Me has been developed for selectivity against COX-1 and showed high and fast brain uptake in the early phase, whereas [^11^C]PK11195 uptake gradually increased and remained for 14 days in a head-to-head comparison study in an LPS-induced inflammation model [63]. Subsequent immunohistochemistry experiments showed that the distribution of activated microglia initially increased because of the expression of COX-1 but not COX-2. In a comparative study with [^11^C]PIB PET, brain uptake increased in the order of healthy controls, mild cognitive impairment (MCI), and AD, however, no significant difference was identified in amyloid-positive [^11^C]KTP-Me PET (average peak cortical standard uptake value: healthy control = 2.3, MCI = 1.6, and AD = 2.1) (Fig. 2. [64]). [^11^C]PS13 presented specific binding for COX-1 in most organs, including the spleen, those in the gastrointestinal tract, and kidneys with abundant COX-1 expression; however, [^11^C]MC1 showed low selective binding for COX-2 in major organs, except for the ovaries [65]. In a recent study involving healthy human subjects, Choi et al. demonstrated that [^11^C]PS13 selectively binds to COX-1 and measured the in vivo potency of nonsteroidal anti-inflammatory drugs against COX-1 [66].

### P2X7 Receptor

The P2X7 receptor is a member of the ATP-gated ion channel purinergic P2X receptor family and is highly expressed in microglia, oligodendrocytes and astrocytes [67]. [^11^C]JNJ54173717 has high affinity for P2X7 (IC_50_ values= 7.6 and 4.2 nM for rat and recombinant human P2X7 orthologs, respectively) and selectively binds to the hP2X7 in nonhuman primates [68]. However, [^11^C]JNJ54173717 PET presented no significant differences in binding between healthy subjects and patients with PD because of a genetic polymorphism (rs3751143) [69]. [^18^F]JNJ64413739 PET displayed increased binding in LPS-treated rats [70]; the radiotracer showed a low test-retest variability (10.7%) and reasonable radiation dosimetry (average effective dose = 22 μSv/MBq) in healthy subjects [71], yet no studies on patients have been reported to date.

### Colony Stimulating Factor 1 (CSF-1) Receptor

Colony stimulating factor-1 (CSF-1) receptors are predominantly expressed in microglia [72]. Although AZ683 has a high affinity for CSF-1R (Ki = 8 nM) and >250 times more selectivity than other kinases, [^11^C]AZ683 PET presented very low brain uptake (< 0.5 SUV) in normal rodents and nonhuman primates [73]. [^11^C]CPPC showed reasonable binding and selectivity to CSF-1R based on findings from preclinical experiments (increment of specific binding: 59% and 120% in LPS-induced mice and baboons, respectively, and 31% in AD mice) and in vitro postmortem autoradiography studies (selectivity baseline/blockade ratio: 2.7) [74]. However, a subsequent study using [^3^H]CPPC showed low specific and off-target binding to many kinase targets [75].

## Imaging Astrocyte

### Imidazole-2 Binding Site (I_2_BS)

The imidazole-2 biding site (I_2_BS) is involved in the regulation of glial fibrillary acidic proteins and is expressed in the mitochondrial membranes of astrocytes. [^11^C]FTIMD showed specific binding to I_2_BS in rodent and nonhuman primate brains, but remaining radioactivity in the pretreatment experiment was approximately 66–75% of baseline, indicating moderate non-specific binding [76, 77], possibly due to the improper specific activity. To overcome this issue, Kawamura et al. developed [^11^C]FTIMD, which possesses ultra-high specific activity (>2000 GBq/μmol), generating a 17–34% decrease in non-specific binding [78]. Kawamura et al. additionally synthesized [^11^C]metrazoline, [^18^F]FEBU, and [^11^C]TEIMD as new radiotracers for I_2_BS. [^11^C]metrazoline and [^18^F]FEBU showed highly specific binding to I_2_BS, but [^11^C]TEIMD had poor brain permeability [79, 80].

**Fig. 3:**
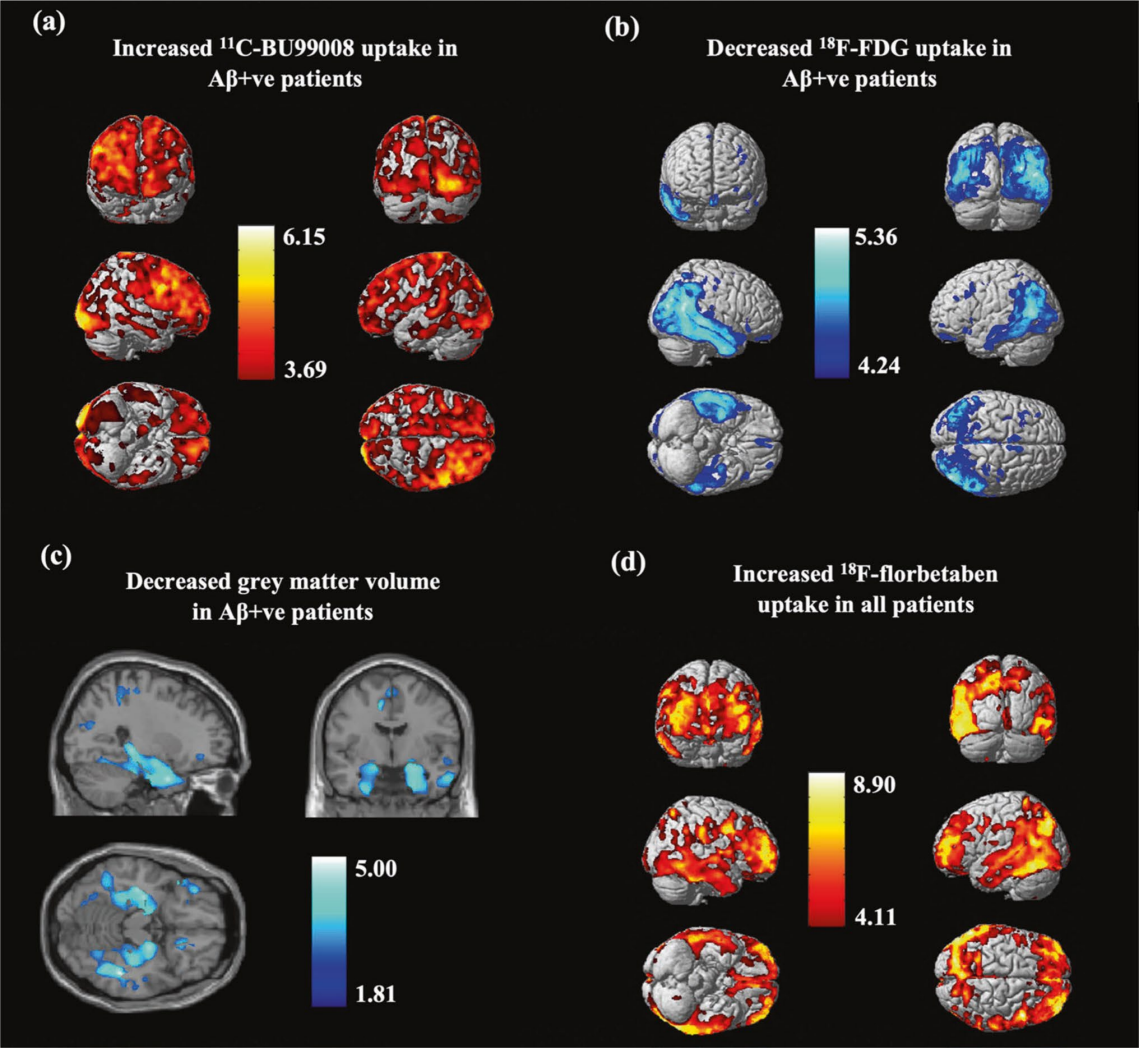
Relationship between [^11^C]BU99008, [^18^F]FDG, gray matter volume and [^18^F]florbetaben in AD patients. This research was originally published in *Mol Psychiatry*. Livingston NR, Calsolaro V, Hinz R, Nowell J, Raza S, Gentleman S, et al. Relationship between astrocyte reactivity, using novel ^11^C-BU99008 PET, and glucose metabolism, grey matter volume and amyloid load in cognitively impaired individuals. Mol Psychiatry. 2022;27:2019-29. http://creativecommons.org/licenses/by/4.0/

Tyache et al. developed [^11^C]BU99008, which has a high specific binding to I_2_BS with a test-retest variability of 15–25% in healthy subjects [81]. In PD, [^11^C]BU99008 PET demonstrated an increased I_2_BS binding in patients with early PD but decreased I_2_BS expression in patients with moderate and advanced PD [82]. In a subsequent study, Mohamed et. al. reported that the binding of [^11^C]BU99008 did not differ between patients with MDD and healthy controls [83]. In a study of cognitively impaired subjects with MCI or probable AD, [^11^C]BU99008 binding was elevated compared to healthy controls [84]. In an AD study using [^11^C]BU99008, astrocyte activity increased in the early stages of low amyloid burden and decreased in the advanced stages of high amyloid burden. In statistical parametric mapping, [^11^C]BU99008 was positively associated with amyloid PET and negatively associated with gray matter volume and glucose PET (Fig. 3. [85]).

### Monoamine Oxidase B

Monoamine oxidase B (MAO-B) plays a prominent role in the degradation of monoamine neurotransmitters, and abnormally high levels of MAO-B activity are associated with various brain diseases. MAO-B is present at low concentrations in microglia and oligodendrocytes and is mainly expressed in the outer mitochondrial membrane of astrocytes [86].

[^11^C]D-deprenyl showed fast clearance, whereas [^11^C]L-deprenyl was retained within the MAO-B-rich regions. Although [^11^C]L-deprenyl exhibited a much higher brain uptake than [^11^C]D-deprenyl, its fast and irreversible binding to MAO-B hinders kinetic analysis [87]. Because carbon-hydrogen dissociation is faster than carbon-deuterium dissociation, the so-called kinetic isotope effect, the same group introduced [^11^C]L-deprenyl-D_2_ ([^11^C]DED) by substituting hydrogen with deuterium in deprenyl [88].

[^11^C]SL25.1188 was developed as a reversible PET tracer for MAO-B and showed high brain permeability and good test-retest reproducibility (12%) in initial clinical trials evaluating healthy subjects [89]. Moriguci et al. reported that the binding of [^11^C]SL25.1188 was significantly increased by approximately 25% in the prefrontal cortex of patients with a major depressive episode [90]. For patients with PTSD, [^11^C]SL25.1188 PET showed an 8–17% reduced availability across the brain regions [91]. Additionally, the total distribution volume of [^11^C]SL25.1188 in traumatic brain injury was considerably higher in the prefrontal cortex compared to the control group [92].

**Fig. 4:**
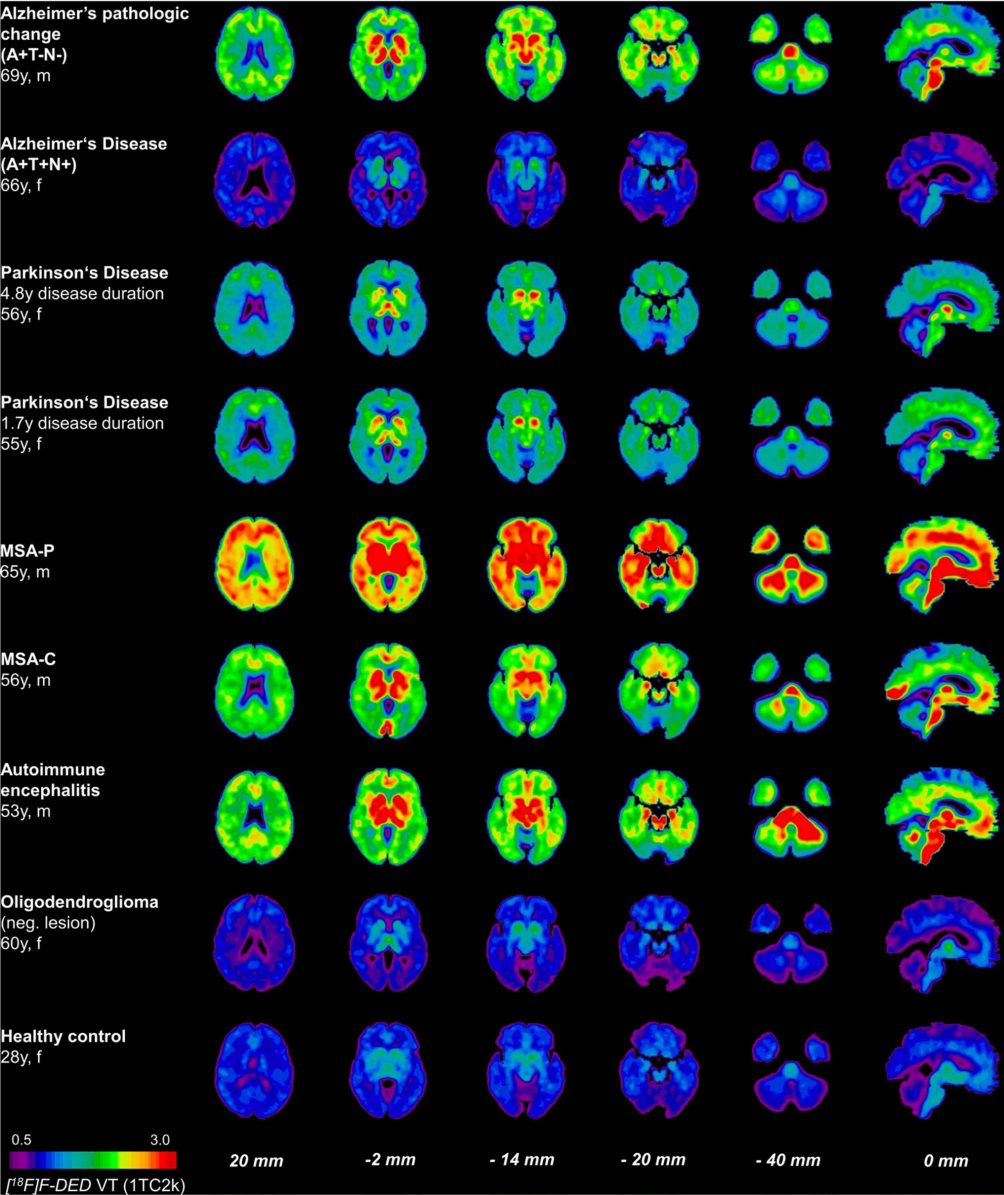
[^18^F]F-DED PET images in various neurodegenerative diseases. This research was originally published in *J Neuroinflammation*. Ballweg A, Klaus C, Vogler L, Katzdobler S, Wind K, Zatcepin A, et al. [^18^F]F-DED PET imaging of reactive astrogliosis in neurodegenerative diseases: preclinical proof of concept and first-in-human data. J Neuroinflammation. 2023;20:68. http://creativecommons.org/licenses/by/4.0/

To increase the clinical applicability of radiotracers, [^18^F]F-DED and [^18^F]FSL25.1188 were developed substituting C-11 by F-18. [^18^F]F-DED PET presented an age-dependent increase in binding in an AD animal model (i.e., APP/PS2), and the PET signals in the hippocampus and thalamus positively correlated with MAO-B expression in glial fibrillary acidic protein (GFAP)-positive astrocytes (R > 0.70). In an initial clinical study that recruited various patients and healthy subjects, [^18^F]F-DED uptake patterns were consistent with MAO-B expression in neurological diseases, as follows: AD without tau and neurodegeneration (A+T-N-), high cortical binding; AD with tau and neurodegeneration (A+T+N+), relatively low cortical binding; PD, moderate global binding with enhanced signal in the basal ganglia; multiple system atrophy, strong cortical binding; and autoimmune encephalitis, strong cerebellar peduncles binding (Fig. 4. [93]). A PET imaging study of [^18^F]FSL25.1188 in nonhuman primates showed a high brain uptake and regional distribution in accordance with MAO-B expression [94].

**Fig. 5:**
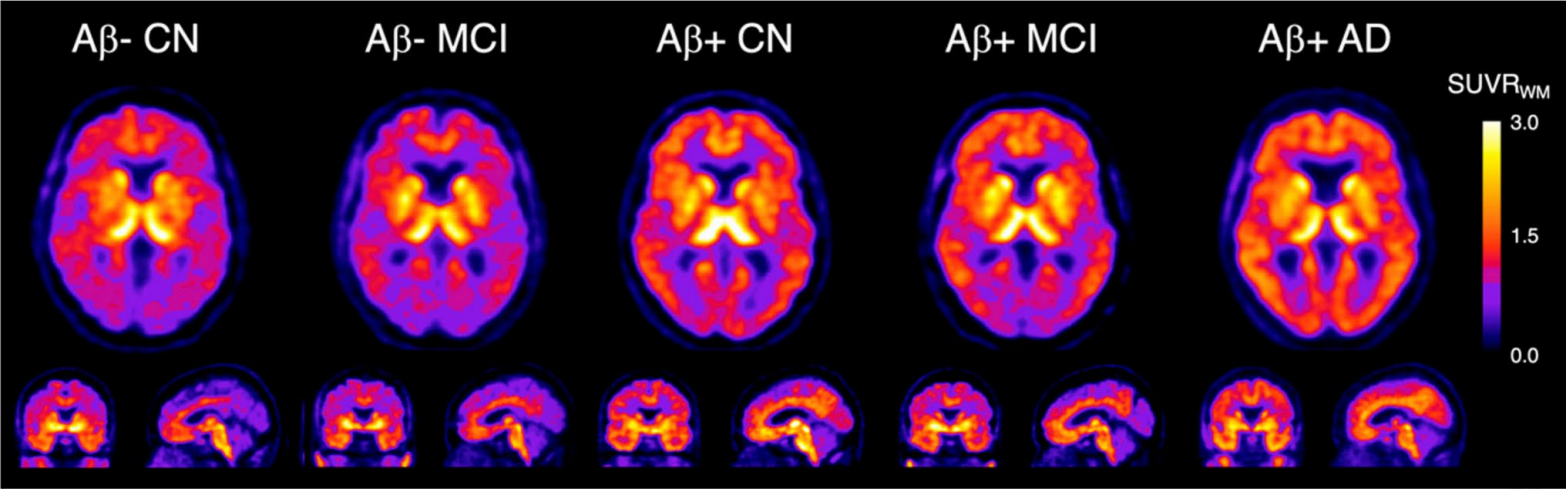
[^18^F]SMBT-1 PET images in AD and cognitive normal subject. This research was originally published in JNM. Villemagne VL, Harada R, Dore V, Furumoto S, Mulligan R, Kudo Y, et al. Assessing Reactive Astrogliosis with ^18^F-SMBT-1 Across the Alzheimer Disease Spectrum. J Nucl Med. 2022;63:1560-9. © SNMMI

In vitro autoradiography of postmortem experiments showed that [^18^F]SMBT-1 possessed a higher specific binding in brain sections of patients with AD than in the control group, and this result was reproduced by MAO-B immunohistochemistry [95]. In a preliminary clinical study, [^18^F]SMBT-1 binding highly correlated with amyloid but not with tau burden. Patients positive for Aβ with normal cognition and those with MCI presented a significantly higher regional binding of [^18^F]SMBT-1 (Fig. 5, [96]). Additionally, Chatterjee et al. have demonstrated that plasma GFAP was associated with regional [^18^F]SMBT-1 signaling in patients with AD [97].

## Discussion

The development of PET tracers for imaging-reactive gliosis based on microglia and astrocytes started in the 1980s, and research and development are currently underway. PET tracers targeting TSPO have been the most actively studied and recent results from MAO-B have been reported. However, no significant differences were found between patient and healthy control groups in clinical trials for CB_2_, COX, and P2X7 [52, 63, 68]. Moreover, the use of [^11^C]CPPC for CSF-1R has the disadvantage of off-target binding [74]. Among the PET tracers developed for the diagnosis of neuroinflammation, the most promising are [^11^C]BU99008, [^18^F]SMBT-1, and [^11^C]SL25.1188 for MAO-B, as such tracers reversible binding to I_2_BS or MAO-B in astrocytes and high permeability to the brain (SUV > 7). However, the clinical efficacy of these PET tracers should be proven through further studies.

In an LPS-based neuroinflammation study by Shukuri et al., COX PET using [^11^C]KTP-Me showed rapid uptake in the inflammatory region and rapid excretion, allowing low non-specific binding; however, TSPO PET using [^11^C]PK11195 displayed relatively slow uptake and maintained the signal in the lesion site, indicating high non-specific binding [62]. These results demonstrate the high nonspecific binding of [^11^C]PK11195 and suggest that other targets are more suitable than TSPO as biomarkers for evaluating neuroinflammation. To clarify this, a comparative study between a third-generation TSPO PET tracer with less nonspecific binding and other target-based PET tracers is warranted.

**Fig. 6:**
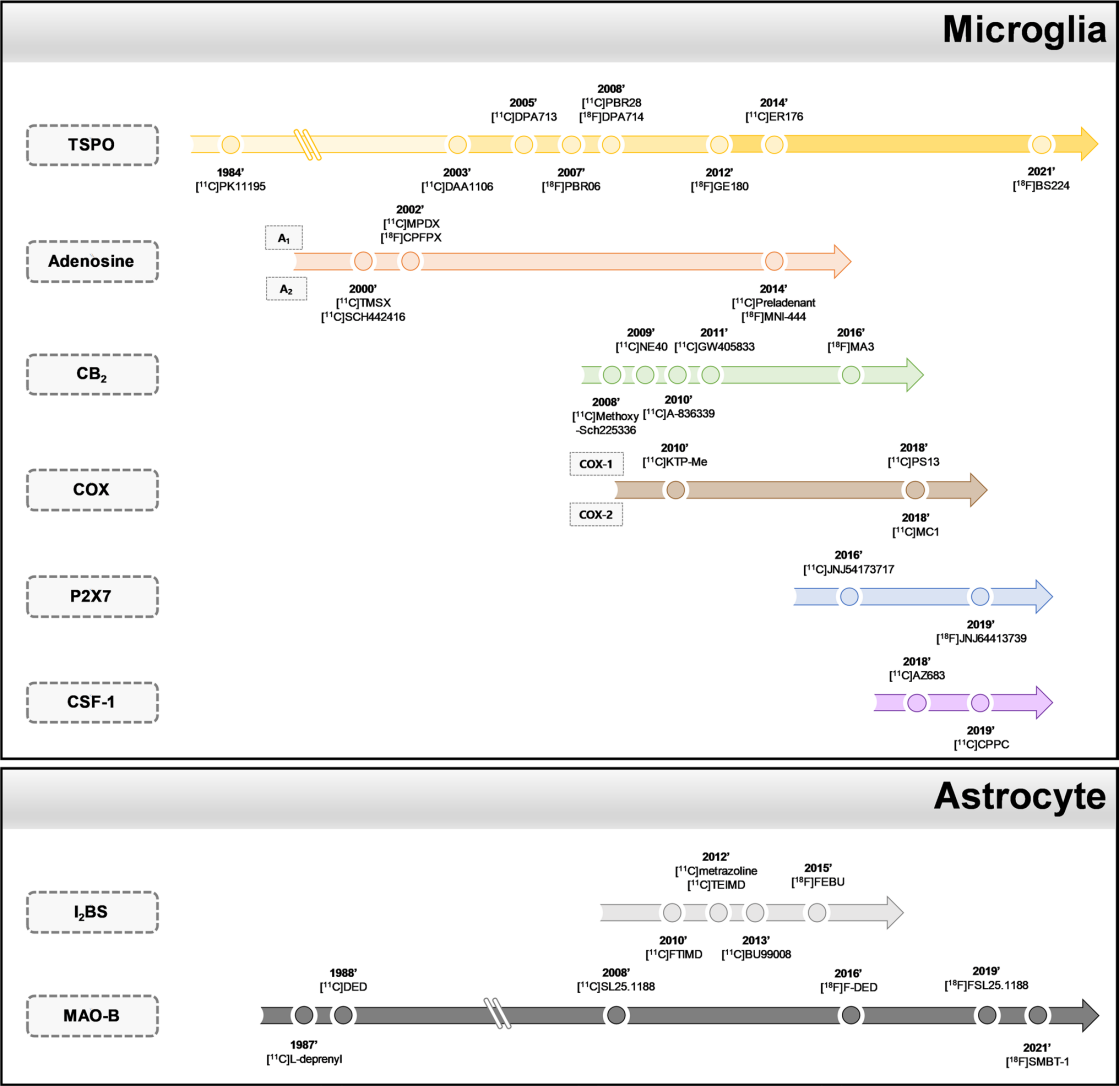
Development trends of PET Tracers for evaluating neuroinflammation since 2010

Biomarker-based PET tracers for microglia and astrocytes development began with TSPO and MAO-B and proceeded in the order of adenosine, CB_2_, COX, P2X7, CSF-1, and I_2_BS (Fig. 6, Table 1) [17, 33, 35, 46, 51, 53, 54, 58–60, 63, 65, 68–71, 73, 74, 76, 77, 79, 80, 87–89, 93–95, 103–161]. Examining clinical trials employing PET tracers conducted after 2014, specific studies on patients, except for healthy subjects, are fewer than those published during the entire drug development period. Most clinical studies using the developed PET tracers vary depending on the biomarkers, however, overall, they have been mainly conducted in North America (US, Canada), Europe (Germany, Belgium, United Kingdom), and Japan. The number of applications for AD and PD was the highest, and studies on degenerative brain diseases, such as neuropsychiatric disorders, MS, and multiple system atrophy, were limited to one or two (Table 2) [29, 31–33, 46, 47, 53, 64, 66, 69, 71, 81, 82, 84, 85, 90, 91, 93, 97, 141, 145, 147, 150, 154, 162–164].

**Table 1:** Developmental status of PET tracers for imaging neuroinflammation

**Biomarker**	**Tracer**	**Developmental status**				**references**
		**Radiochemical**	**Pre-clinical**		**Clinical**	
			**Rodent**	**Non-human primate**		
TSPO	[^11^C]PK11195	√	√	√	√	[103–106]
	[^11^C]DAA1106	√	√	√	√	[17, 107–109]
	[^11^C]DPA713	√	√	√	√	[110–113]
	[^18^F]PBR06	√	√	√	√	[114–116]
	[^11^C]PBR28	√	√	√	√	[117–120]
	[^18^F]DPA714	√	√	√	√	[111, 121, 122]
	[^18^F]GE180	√	√	-	√	[120, 123, 124]
	[^11^C]ER176	√	√	√	√	[33, 125–127]
	[^18^F]BS224	√	√	-	-	[35]
Adenosine	[^11^C]TMSX	√	√	√	√	[128, 129]
	[^11^C]SCH442416	√	√	√	√	[130–132]
	[^11^C]MPDX	√	√	√	√	[133, 134]
	[^18^F]CPFPX	√	√	√	√	[133, 135–137]
	[^11^C]Preladenant	√	√	√	√	[138–140]
	[^18^F]MNI-444	√	-	√	√	[46, 141, 142]
CB_2_	[^11^C]Methoxy-Sch225336	√	-	-	-	[51]
	[^11^C]NE40	√	√	√	√	[53, 143]
	[^11^C]A-836339	√	√	-	-	[54]
	[^11^C]GW405833	√	√	-	-	[58]
	[^18^F]MA3	√	√	√	-	[59, 60]
COX	[^11^C]KTP-Me	√	√	-	√	[63, 144, 145]
	[^11^C]PS13	√	-	√	√	[146, 147]
	[^11^C]MC1	√	-	√	-	[65, 148]
P2X7	[^11^C]JNJ-54173717	√	√	√	√	[68, 69]
	[^18^F]JNJ-64413739	√	√	√	√	[70, 71, 149]
CSF-1	[^11^C]AZ683	√	√	√	-	[73]
	[^11^C]CPPC	√	√	√	√	[74, 150]
I_2_BS	[^11^C]FTIMD	√	√	√	-	[76, 77]
	[^11^C]metrazoline	√	-	-	-	[80]
	[^11^C]TEIMD	√	-	-	-	[80]
	[^11^C]BU99008	√	√	√	√	[151–154]
	[^18^F]FEBU	√	√	-	-	[79]
MAO-B	[^11^C]L-deprenyl	√	-	-	√	[87, 155]
	[^11^C]DED	√	√	-	√	[88, 156, 157]
	[^11^C]SL25.1188	√	-	√	√	[89, 158, 159]
	[^18^F]DED	√	√	√	√	[93, 160]
	[^18^F]FSL25.1188	√	-	√	-	[94]
	[^18^F]SMBT-1	√	-	-	√	[95, 161]

**Table 2:** Representative PET tracers for imaging neuroinflammation undergoing clinical trials since 2010

**Biomarker**	**Tracer**	**Affiliation**	**Subjects**	**references**
TSPO	[^11^C]ER176	*National Institute of Mental Health, USA*	HC (2016)	[33]
		*Nantz National Alzheimer Center, USA*	HC (2019)	[29]
	[^18^F]GE180	*Imperial College London, UK*	HC (2016)	[162, 163]
		*Ludwig-Maximilians University Munich, Germany*	HC, RRMS (2018)	[31]
		*University of California, USA*	HC, CCM (2023)	[32]
Adenosine	[^18^F]MNI-444	*Molecular NeuroImaging, LLC, USA*	HC (2015)	[46]
	[^11^C]Preladenant	*Tokyo Metropolitan Institute of Gerontology, Japan*	HC (2017), HC, PD (2018), PD (2022)	[47, 141, 164]
CB_2_	[^11^C]NE40	*University Hospital Leuven, Belgium*	HC, AD (2016)	[53]
COX	[^11^C]KTP-Me	*Center for Life Science Technologies RIKEN, Japan*	HC (2014), HC, PiB, MCI, AD (2016)	[64, 145]
	[^11^C]PS13	*National Institute of Mental Health, USA*	HC (2020), (2023)	[66, 147]
P2X7	[^11^C]JNJ-54173717	*University Hospital Leuven, Belgium*	HC, PD (2019)	[69]
	[^18^F]JNJ-64413739	*University Hospital Leuven, Belgium*	HC (2019)	[71]
CSF-1	[^11^C]CPPC	*Johns Hopkins Medical Institutions, USA*	HC (2022)	[150]
I_2_BS	[^11^C]BU99008	*Imperial College London, UK*	HC (2018), HC, MCI, AD (2021), (2022)	[81, 84, 85, 154]
		*King’s College London, UK*	HC, PD (2019)	[82]
MAO-B	[^11^C]SL25.1188	*University of Toronto, Canada*	HC (2014), HC, MDD (2019), HC, PTSD (2022)	[90, 91]
	[^18^F]SMBT-1	*Tohoku Medical and Pharmaceutical University, Japan*	HC, MCI, AD (2022)	[161]
		*University of Pittsburgh, USA*	HC, MCI, AD (2022)	[97]
	[^18^F]DED	*University Hospital of Munich, Germany*	HC, AD, PD, MSA, AIE, OG (2023)	[93]

(HC : healthy controls, RRMS : relapsing-remitting multiple sclerosis, CCM : cerebral cavernous malformations, PD : Parkinson’s disease, AD : Alzheimer’s disease, PiB : Pittsburgh compound-B, MCI : mild cognitive impairment, MDD : major depressive disorder, PTSD : posttraumatic stress disorder, MSA : multiple system atrophy, AIE : autoimmune encephalitis, OG : oligodendroglioma)z

Although PET tracers developed thus far have shown excellent pharmacokinetic characteristics in the preclinical stage (such as high affinity and selectivity, and metabolic stability), most clinical studies did not identify significant differences between patients and control groups or showed inconsistent results. The main issues in developing a successful PET tracer that can be used in clinical settings are as follows. First, previous studies have mainly used rodents, whereas glial cells have species-specific differences. Human microglia and astrocytes are significantly different from those of rodents in terms of morphology, gene expression, and function [98–102]. Therefore, despite the limitations of using postmortem human microglia and astrocytes, research on human in vitro models is indispensable to verify their efficacy. Second, the reason for the lack of a significant differences between the patient and control groups may be that the affinity of the PET tracer is not high enough to detect changes in low concentrations of biomarkers. [^11^C]PS13 for COX-1 and [^11^C]MC1 for COX-2 had similar affinities at a level of 1nM, yet only [^11^C]PS13 possessed reasonable brain uptake. To address this, Kim et al. suggested that the constituent density of COX-2 was much lower than that of COX-1, which indicates that the PET tracer for COX-2 should have a high affinity at the pM level. Additionally, Atili et al. suggested that the reason for the failure of the blockade experiment for [^11^C]MA3 was that the expression of CB_2_ receptors was too low at normal levels. Third, the degree of neuroinflammation does not appear to have a one-dimensional proportional relationship with the severity of brain disease pathology. That is, although neuroinflammation is closely related to various brain diseases, the density of activated glial cells tends to decrease as the disease progresses. Therefore, PET-identified gliosis may not be different from the healthy control group depending on the severity of the disease in the participating patient. For instance, studies on AD have shown that the density of reactive glial cells increases before tau pathology (A+T-N-) accumulates, but decreases after tauopathies are severed. Some researchers have suggested that neuroinflammation occurs faster than amyloid pathology during the course of AD. However, to the best of our knowledge, studies related to temporal changes over time are limited, and further research is required to address this issue.

Neuroinflammation in brain diseases may be caused by the interaction between microglia and astrocytes rather than by the activation of the two alone. Most studies conducted to date have focused on in vivo changes in one biomarker and used it in a specific disease stage rather than in life-cycle studies. However, if further research using PET tracers for each biomarker is conducted, the role of temporal and causal interactions between the two glial components could also be identified. In addition, future studies should identify and verify reference issues that are specific to diseases. For AD, the cerebellum is proposed to be a pseudo-reference tissue, as the disease does not appear considerably in the cerebellum until it progresses. However, if a pathology affects the cerebellum, such as in multiple system atrophy or autoimmune encephalitis, another reference region should be identified. Lastly, determining the optimal scan time window after tracer injection through a comparative study of arterial input function-based and reference tissue-based dynamic scans could reduce the burden on subjects and contribute to clinical research.

## Conclusion

Non-invasive in vivo evaluation of neuroinflammation based on activated glial cells is expected to play an important role in the course of neurodegenerative and psychiatric disorders, influencing the development of therapeutic drugs as well as determining disease severity and treatment methods. Despite the development of PET tracers based on several biomarkers, only a few drugs that can be used in clinical practice currently exist.

If research is conducted in the direction of 1) verifying validity by conducting multicenter clinical studies or follow-up studies using previously developed candidate PET tracers and 2) developing new PET tracers to overcome the difficulties highlighted in this review, promising PET radiopharmaceuticals that can be used in clinical practice could be available in the near future.

## Data Availability

Data sharing not applicable to this article as no datasets were generated in the current study.
