# Correction to: The Development Status of PET Radiotracers for Evaluating Neuroinflammation

**DOI:** 10.1007/s13139-024-00880-3

**Published:** 2024-08-27

**Authors:** Namhun Lee, Jae Yong Choi, Young Hoon Ryu

**Affiliations:** 1 Division of Applied RI, Korea Institute of Radiological & Medical Sciences, 75 Nowon‑ro, Nowon‑gu, Seoul, 01812 Korea; 2 Radiological and Medico‑Oncological Sciences, University of Science and Technology (UST), Seoul, Korea; 3 Department of Nuclear Medicine, Gangnam Severance Hospital, Yonsei University College of Medicine, Seoul, Korea

**Correction to Nuclear Medicine and Molecular Imaging (2024) 58:160–176**

10.1007/s13139-023-00831-4

A correction to this paper has been published: 10.1007/s13139-023-00831-4.

The article The development status of PET radiotracers for evaluating neuroinflammation, written by Namhun Lee, Jae Yong Choi and Young Hoon Ryu, was originally published Online First without Open Access. After publication in volume 58, issue 4, pages 160–176 the author decided to opt for Open Choice and to make the article an Open Access publication. Therefore, the copyright of the article has been changed to © The Authors, 2024 and the article is forthwith distributed under the terms of the Creative Commons Attribution (CC BY) license 4.0 International License, which permits use, sharing, adaptation, distribution and reproduction in any medium or format, as long as you give appropriate credit to the original author(s) and the source, provide a link to the Creative Commons licence, and indicate if changes were made. The images or other third party material in this article are included in the article’s Creative Commons licence, unless indicated otherwise in a credit line to the material. If material is not included in the article’s Creative Commons licence and your intended use is not permitted by statutory regulation or exceeds the permitted use, you will need to obtain permission directly from the copyright holder. To view a copy of this licence, visit http://creativecommons.org/licenses/by/4.0.

The original article has been corrected.

